# Frühe Sprachleistungen im Fragebogen ELFRA: Auswertung multizentrischer Daten von Kindern nach bilateraler Cochleaimplantatversorgung

**DOI:** 10.1007/s00106-024-01489-w

**Published:** 2024-06-11

**Authors:** Cynthia Glaubitz, Rainer Beck, Tim Liebscher, Antje Aschendorff, Kerstin Kreibohm-Strauß, Dominique Kronesser, Yvonne Seebens, Barbara Streicher, Stefanie Kröger

**Affiliations:** 1 Hals-Nasen-Ohrenklinik, Kopf- und Halschirurgie, Cochlear-Implant-Centrum CICERO, Uniklinikum Erlangen, FAU Erlangen-Nürnberg, Erlangen, Deutschland; 2 Klinik und Poliklinik für Hals-Nasen-Ohrenheilkunde, Sektion Implant Centrum (ICF), Universitätsklinikum Freiburg, Medizinische Fakultät, Albert-Ludwigs-Universität Freiburg, Freiburg, Deutschland; 3 Cochlear Implant Centrum Wilhelm Hirte, Hannover, Deutschland; 4 Klinik und Poliklinik für Hals-Nasen- und Ohrenheilkunde, Sächsisches Cochlear Implant Centrum (SCIC), Universitätsklinikum Carl Gustav Carus, Dresden, Deutschland; 5 Cochlear Implant Center (CIC) Rhein-Main des HSF gGmbH, Friedberg, Deutschland; 6 HNO-Klinik, Cochlear Implant Centrum Köln (CIK), Uniklinik Köln, Köln, Deutschland

**Keywords:** ACIR, Referenzwerte, Vorsprachliche Kompetenzen, Schwerhörigkeit, Gesten, ACIR, Reference values, Prelingual skills, Hearing loss, Gestures

## Abstract

**Hintergrund:**

Eine sehr frühe bilaterale Cochleaimplantat(CI)-Versorgung ist bei Kindern heute etablierter Standard. Die Erfassung präverbaler und verbaler Kompetenzen in sehr frühen Entwicklungsphasen gewinnt damit an Bedeutung. Diagnostisch erhobene Leistungsdaten werden für Kinderkohorten in Bezug auf deren Lebensalter (LA) und Höralter (HA) evaluiert und dargestellt.

**Methodik:**

Die vorliegende Studie als Teil einer retrospektiven Multizenterstudie inkludierte 4 Kinderkohorten (*n* = 72–233) bilateral CI-versorgter Kinder ohne Zusatzbeeinträchtigungen und untersuchte deren Ergebnisse in den Elternfragebögen zur Früherkennung von Risikokindern (ELFRA‑1 bzw. ELFRA-2) unterteilt nach LA- und HA-bezogener Diagnostik. Die Daten wurden zudem hinsichtlich Mono‑/Bilingualität und CI-Versorgungsalter analysiert.

**Ergebnisse:**

Die verbalen Leistungen fielen bezogen auf das LA geringer aus als bezogen auf das HA. Die präverbalen Kompetenzen waren weitestgehend LA-adäquat. Kinder mit bi-/multilingualem Spracherwerb zeigten signifikant geringere Leistungen. Für den ELFRA‑2 waren die LA-bezogenen verbalen Leistungen negativ mit dem CI-Versorgungsalter korreliert.

**Schlussfolgerung:**

Bei früher CI-Versorgung sollte das LA als Bezugsmaß in der Diagnostik dem HA vorgezogen werden, um den individuellen Leistungsstand exakter zu erfassen und Ergebnisverzerrungen zu vermeiden. Die ermittelten Perzentile eignen sich begrenzt als allgemeingültige Referenzwerte für die individuelle Leistungseinschätzung bilateral CI-versorgter Kinder. Weitere Multizenterstudien sind anzustreben.

## Abstract (en)

**Background:**

Very early bilateral cochlear implant (CI) provision is today’s established standard for children. Therefore, the assessment of preverbal and verbal performance in very early stages of development is becoming increasingly important. Performance data from cohorts of children were evaluated and presented based on diagnostic assessment using chronological age (CA) and hearing age (HA).

**Methods:**

The present study, as part of a retrospective multicentre study, included 4 cohorts (*N* = 72–233) of children with bilateral CI without additional disabilities. Their results in the German parent questionnaires *Elternfragebögen zur Früherkennung von Risikokindern*(ELFRA‑1 and ELFRA-2) subdivided for CA and HA were statistically analysed. The data were also analysed in terms of mono-/bilingualism and age at CI provision.

**Results:**

Overall, verbal performance in relation to CA was lower than in relation to HA. Preverbal skills were largely CA appropriate. Children with bi-/multilingual language acquisition performed significantly lower. Verbal performance in ELFRA‑2 referenced to CA was negatively correlated with age at CI provision.

**Conclusion:**

In the case of early CI provision, CA should be the preferred reference mark in preverbal and verbal assessment in order to obtain exact individual performance levels and avoid bias in results. The percentiles determined are of limited use as generally valid reference values to which the individual performance of bilaterally implanted children could be compared. Further multicentre studies should be initiated.

## Frühe Phasen der Entwicklung

Mit der Cochleaimplantat(CI)-Versorgung von hochgradig schwerhörigen bzw. gehörlosen Kindern in sehr jungem Alter ist die Betrachtung sehr früher Phasen der kindlichen Sprachentwicklung von hoher Relevanz. Zumeist werden die Kompetenzen der CI-versorgten Kinder mit den Daten normalhörender Normierungsstichproben etablierter Erhebungsverfahren verglichen. Ein Vergleich der individuellen Leistung mit jenen von CI-versorgten Kinderkohorten ist zum aktuellen Zeitpunkt nicht möglich, da keine entsprechenden Verfahren bzw. Normierungen im deutschsprachigen Raum vorliegen.

### Frühestmögliche CI-Versorgung

Eine CI-Versorgung bei kongenital gehörlosen Kindern wird heute in möglichst jungem Alter der Kinder durchgeführt, um das auditive Deprivationsrisiko so gering wie möglich zu halten. Zu diesem Zweck sehen aktuell vorliegenden Leitlinien und Regularien zur CI-Versorgung [8, 9] bei Kindern auch einen präoperativen Hörgeräte-Trageversuch vor sowie eine bilaterale Implantation möglichst innerhalb des ersten Lebensjahres. Erwiesenermaßen zeigen sich mit diesem Prozedere günstigere Entwicklungsverläufe in Bezug auf die kindlichen Hör- und Sprachleistungen [7, 15]. Entsprechende Untersuchungen zielten in der Vergangenheit häufig auf komplexere, audiometrisch messbare Hörleistungen im Sinne der Sprachperzeption sowie explizit auf lautsprachlich expressive Kompetenzen [2] ab. Mit zunehmend jüngerem Alter der CI-versorgten Kinder rückten jedoch auch die frühen Phasen der Entwicklung in den Fokus wissenschaftlicher Studien [1, 11].

### Bedeutung präverbaler kommunikativer Kompetenzen

Bei Betrachtung von sehr frühen entwicklungspsychologischen Prozessen sind in Bezug auf den Spracherwerb neben den basalen sensorisch-auditiven Fähigkeiten auch weitere präverbale Kompetenzbereiche in Augenschein zu nehmen [21, 29]. Hierzu zählen u. a. die sensomotorischen Fähigkeiten, welche im linguistischen Sinne für die spätere Sprechmotorik und gleichermaßen für den Erwerb und die Verwendung von gestischen und mimischen Ausdrucksmitteln bedeutsam sind. Kinder wenden bereits in präverbalen Phasen deiktische Gesten an, um sich zu äußern und mitzuteilen. Diese Fertigkeiten sind im Rahmen der intermodalen Wahrnehmungsentwicklung wichtig, um weitere kommunikationsrelevante Entwicklungsschritte – wie die Ausbildung von Imitationsverhalten, Triangulierung, Interaktionsfähigkeit und Situationsverständnis – entwickeln zu können. Auf diesen Vorausläuferfertigkeiten baut in der Folge der Erwerb lautsprachlicher rezeptiver (Sprachverständnis) und produktiver Kompetenzen (Lautierungen, Wortproduktion, Mehrwortäußerungen) auf.

Bestimmte präverbale Kompetenzbereiche sind unabhängig von der Spracherwerbsmodalität, sodass auch für bi-/multilingual oder bimodal mit Gebärdensprache aufwachsende Kinder die präverbale Entwicklung ein Indikator für die Lautsprachentwicklung ist. Frühere Studien mit CI-versorgten Kindern zeigten, dass sich bi-/multilinguale Erwerbsbedingungen ungünstig auf lexikalische und syntaktische Lautsprachkompetenzen auswirken [16, 27]. Befunde für die Vorausläuferfertigkeiten Feinmotorik und Gestik bei CI-versorgten Kindern finden sich bisher nur vereinzelt [20]. Insgesamt besteht hinsichtlich der Mehrsprachigkeit weiterer Forschungsbedarf, bei dem die Besonderheiten in der Anwendung sprachdiagnostischer Tests bei bi-/monolingualen Kindern [14, 25] berücksichtigt werden.

### Diagnostik früher Kompetenzen bei CI-Versorgung

Diagnostische Verfahren, die den Spracherwerb bei sehr jungen Kindern untersuchen, sollten demnach im Idealfall verbale wie präverbale Basiskompetenzen erfassen [6]. Für den deutschsprachigen Raum eignen sich hierfür bei Kindern im Alter von 1 und 2 Jahren die Elternfragebögen zur Früherkennung von Risikokindern (ELFRA) [13]. Dieses standardisierte Verfahren ist zwar anhand normalhörender Kinder normiert, jedoch auch in der Anwendung während der postoperativen Rehabilitationsphase bei CI-versorgten Kindern etabliert und empfohlen [6, 8, 19]. Der direkte Vergleich von CI-versorgten Kindern mit normalhörenden Gleichaltrigen einer Normierungsstichprobe birgt jedoch Risiken hinsichtlich der Ergebnisinterpretation, da für die beiden Vergleichsgruppen unterschiedliche Bedingungen für die Sprachentwicklung bestehen. Aufgrund dieses Dilemmas wird häufig im Rahmen der Diagnostik das sog. Höralter dem Lebensalter des Kindes als Bezugsmaß vorgezogen. Das Höralter bezieht sich auf die individuelle Hörerfahrung mit CI und ist definiert als der Zeitabstand zur initialen Aktivierung des ersten CI-Systems bei sequenzieller bzw. beider CI-Systeme bei simultaner Versorgung. Das Höralter soll v. a. der präoperativen auditiven Deprivationsphase Rechnung tragen. Somit wird das Testergebnis des CI-versorgten Kindes mit einer entsprechend jüngeren Altersgruppe normalhörender Kinder verglichen und davon ausgehend der Leistungsstand beurteilt. Unberücksichtigt bleibt dabei jedoch häufig der präoperative Hörstatus unter einer Versorgung mit Hörgeräten; in dieser Phase kann gut genutztes Restgehör ggf. entwicklungsrelevant sein. Es kann daher bei vielen Kindern zu einer Verzerrung bzw. Überschätzung des tatsächlichen Leistungstands kommen, wenn dieser rein anhand des Höralters beurteilt wird. Dies wird durch Studien gestützt, bei denen unter ausschließlicher Anwendung des Höralters als Bezugsmaß abweichende Verläufe in frühen Spracherwerbsphasen unzureichend erfasst werden konnten [22]. Heute wird empfohlen, die Hör- und Sprachentwicklungsdiagnostik unter Anwendung beider Bezugsmaße vorzunehmen [8].

### Fragestellungen der Studie

Um eine adäquate Einschätzung von verbalen Kompetenzen in frühen Phasen der Sprachentwicklung CI-versorgter Kinder zukünftig zu erleichtern, hat die vorliegende Arbeit zum Ziel, die in den Fragebögen ELFRA erzielten Ergebnisse einer großen Kohorte an CI-versorgten Kindern darzustellen. Zentrale Fragestellungen sind hierbei: Wie stellen sich präverbale und verbale Leistungsbereiche CI-versorgter Kinder im Vergleich zur Normierungsstichprobe des Fragebogenverfahrens dar, differenziert hinsichtlich der Durchführung und Auswertung nach Lebensalter und nach Höralter mit CI? Besteht ein Zusammenhang zwischen den Leistungen und dem CI-Versorgungsalter der Kinder? Können die ermittelten Daten als Referenzwerte für einen Abgleich mit individuellen Testwerten herangezogen werden? Des Weiteren wird geprüft, ob bedeutsame Leistungsunterschiede zwischen Kindern mit mono- versus bi-/multilingualen bzw. bimodalen Spracherwerbsbedingungen bestehen.

## Material und Methoden

Die vorliegende Datenanalyse erfolgte im Rahmen einer retrospektiv angelegten Multizenterstudie der Arbeitsgemeinschaft CI-Rehabilitation (ACIR e. V.) mit Beteiligung 6 großer deutscher CI-Centren [17]. Die in diese Multizenterstudie inkludierten Kinder (*n* = 838) wurden im Zeitraum von 2001 bis 2021 in einer der 6 Einrichtungen umfassend betreut und in diesem Rahmen entwicklungsdiagnostisch mit standardisierten Verfahren untersucht. Die Auswertung aller zusammengetragenen und aufbereiteten Daten erfolgte in zugehörigen Teilstudien, die sich jeweils auf die Analyse der Diagnostikergebnisse einzelner Verfahren konzentrierten. Untersuchungsgegenstand der vorliegenden Arbeit sind die Daten der ELFRA-Fragebögen.

### Stichprobe

In die Multizenterstudie wurden kongenital bilateral gehörlose Kinder eingeschlossen, welche simultan oder sequenziell (maximaler Abstand 12 Monate) bis spätestens zum Zeitpunkt 48 Lebensmonate bilateral mit CI versorgt waren. Weiteres Einschlusskriterium war eine sichergestellte auditive Diskriminationsfähigkeit zum Zeitpunkt der entwicklungsdiagnostischen Erhebungen. Zur Reduzierung von konfundierenden Einflüssen wurden als Ausschlusskriterien das Vorliegen von entwicklungsrelevanten Zusatzbeeinträchtigungen – Kognition und/oder Motorik betreffend; zum Zeitpunkt der Datensammlung vorliegende Diagnose(n) –, Erkrankungen, Syndromen, anatomischen cochleären und neuronalen Dysplasien, unvollständige intracochleäre Elektrodeninsertion oder irreguläre Elektrodenlage festgelegt. Es wurde erfasst, ob der Spracherwerb der Kinder mono-, bi-/multilingual oder bimodal erfolgte.

Aus allen Kindern der Multizenterstudie wurden für die vorliegende Analyse diejenigen Kinder extrahiert, für welche zum einen Datensätze des ELFRA‑1 und zum zweiten Datensätze des ELFRA‑2 vorlagen. Lagen für ein Kind mehrere Daten für eine Fragebogenversion vor, so wurde derjenige Datensatz ausgewählt, welcher zeitlich am nächsten zu den Testzeitpunkten 12 bzw. 24 Monaten lag. Die Tab. 1 zeigt die genauen Stichprobenumfänge und die miterfassten demografischen Variablen, getrennt für die Daten hinsichtlich der Bezugsmaße Lebensalter (LA) und Höralter (HA). Da nicht für alle Kinder Datensätze zu allen Testzeitpunkten vorlagen, variiert der Umfang der einzelnen querschnittlichen Teilstichproben.

**Tab. 1:** Demografische Variablen der Teilstichproben, separiert für die Bezugsmaße und die Fragebogenversionen der Elternfragebögen zur Früherkennung von Risikokindern (ELFRA)

Bezugsmaß	Variablen		ELFRA‑1	EFLRA‑2
Lebensalter	Teilstichproben-Umfang		*n*_LA12_ = 99	*n*_LA24_ = 72
	Geschlecht	Männlich	*n* = 55 (56 %)	*n* = 36 (50 %)
		Weiblich	*n* = 44 (44 %)	*n* = 36 (50 %)
	Spracherwerb	Monolingual	*n* = 74 (75 %)	*n* = 52 (72 %)
		Bi-/multilingual	*n* = 18 (18 %)	*n* = 14 (20 %)
		Bimodal Gebärden	*n* = 7 (7 %)	*n* = 6 (8 %)
	Ätiologie der Hörbeeinträchtigung	Unbekannt oder unklar	*n* = 62 (63 %)	*n* = 45 (62 %)
		Genetisch	*n* = 26 (26 %)	*n* = 20 (28 %)
		Infektiös	*n* = 11 (11 %)	*n* = 7 (10 %)
	CI-Versorgung	Simultan	*n* = 60 (61 %)	*n* = 25 (35 %)
		Sequenziell	*n* = 39 (39 %)	*n* = 47 (65 %)
	CI-Versorgungsalter in Monaten		M = 10,6 (SD = 2,3), Spannbreite 6–17	M = 11,4 (SD = 4,0), Spannbreite 6–24
	Testalter/Lebensalter in Monaten		M = 15,3 (SD = 2,3), Spannbreite 11–18	M = 24,7 (SD = 0,9), Spannbreite 23–26
	Hörerfahrung mit CI zum Testzeitpunkt in Monaten		M = 4,7 (SD = 2,1), Spannbreite 1–11	M = 13,3 (SD = 4,0), Spannbreite 1–18
Höralter	Teilstichproben-Umfang		*n*_HA12_ = 233	*n*_HA24_ = 161
	Geschlecht	Männlich	*n* = 125 (54 %)	*n* = 84 (52 %)
		Weiblich	*n* = 108 (46 %)	*n* = 77 (48 %)
	Spracherwerb	Monolingual	*n* = 157 (67 %)	*n* = 113 (70 %)
		Bi-/multilingual	*n* = 53 (23 %)	*n* = 31 (19 %)
		Bimodal Gebärden	*n* = 23 (10 %)	*n* = 17 (11 %)
	CI-Versorgung	Simultan	*n* = 111 (48 %)	*n* = 71 (44 %)
		Sequenziell	*n* = 122 (52 %)	*n* = 90 (56 %)
	Ätiologie der Hörbeeinträchtigung	Unbekannt oder unklar	*n* = 153 (66 %)	*n* = 108 (67 %)
		Genetisch	*n* = 61 (26 %)	*n* = 36 (22 %)
		Infektiös	*n* = 19 (8 %)	*n* = 17 (11 %)
	CI-Versorgungsalter in Monaten		M = 15,5 (SD = 7,6), Spannbreite 6–45	M = 14,7 (SD = 7,3), Spannbreite 6–47
	Testalter/Höralter in Monaten		M = 12,9 (SD = 1,7), Spannbreite 11–18	M = 24,3 (SD = 1,2), Spannbreite 22–26
	Lebensalter zum Testzeitpunkt in Monaten		M = 28,4 (SD = 7,8), Spannbreite 7–58	M = 39,1 (SD = 7,4), Spannbreite 7–69

*HA12* Höralter 12 Monate, *HA24* Höralter 24 Monate,* LA12* Lebensalter 12 Monate, *LA24* Lebensalter 24 Monate, *M* Mittelwert, *SD* Standardabweichung

### Fragebögen ELFRA-1 und ELFRA-2

Die standardisierten Elternfragebögen zur Früherkennung von Risikokindern (ELFRA) beinhalten 2 Versionen für 2 Alters- bzw. Testzeitpunkte: ELFRA‑1 für 12 Monate und ELFRA‑2 für 24 Monate. Sie erfassen Kompetenzbereiche der frühen Sprachentwicklung, indem anhand von Elternantworten Rohwerte ermittelt werden. Diese werden mit normierten, den sog. kritischen Werten (80 % der Normierungsstichprobe haben diesen Wert erreicht) abgeglichen, woraus sich der individuelle Risikostatus des Kindes ergibt. In Tab. 2 sind die einzelnen Entwicklungsskalen getrennt für die Fragebogenversionen sowie die zugehörigen beurteilungsrelevanten Werte im Detail dargestellt. Unabhängig von mono-/bi-/multilingualem und bimodalem Spracherwerb wurden der ELFRA‑1 und ELFRA‑2 für die deutsche Sprache beurteilt.

**Tab. 2:** Altersbezogene Versionen der standardisierten Elternfragebögen zur Früherkennung von Risikokindern (ELFRA) mit Angabe der jeweils erfassten Entwicklungsskalen und relevanten Werte zur Beurteilung des kindlichen Risikostatus

	Entwicklungsskala	Abkürzung	Maximaler Rohwert	Kritischer Wert
ELFRA‑1	Sprachproduktion	SP	181	7
	Sprachverständnis	SV	171	17
	Gesten	G	30	11
	Feinmotorik	F	13	7
ELFRA‑2	Produktiver Wortschatz	PW	260	50
	Syntax	Syn	47	7
	Morphologie	Mor	16	2

### Testzeitpunkte und Bezugsmaße

In der vorliegenden Studie wurden die Daten des ELFRA‑1 und ELFRA‑2 von 4 voneinander unabhängigen Testzeitpunkten analysiert (in Klammern sind Minimum und Maximum der im Datensatz inkludierten Testzeitpunkte angegeben): LA 12 (11–18) Monate und LA 24 (22–26) Monate sowie HA 12 (11–18) Monate und HA 24 (22–26) Monate. Die Spannweite für die Bezugsmaße im ELFRA‑1 wurde im Vergleich zum ELFRA‑2 größer gewählt, da laut Testmanual eine Befragung mittels des ELFRA‑1 auch zum Lebensalterszeitpunkt 18 Monate durchgeführt werden kann. Statistische Voranalysen ergaben zudem keine signifikanten Leistungsunterschiede in den Skalen des ELFRA‑1, verglichen zwischen den Alterszeitpunkten 12 (Spannbreite 11–14) und 18 (Spannbreite 15–18) Monaten, sodass die Gruppen für die Datenanalyse zusammengelegt wurden. Für die LA-Analysen wurde zudem das Kriterium gesetzt, dass die Kinder zum jeweiligen Testzeitpunkt mindestens einen Monat mit dem ersten CI versorgt waren.

### Datenauswertung

Die statistische Auswertung erfolgte mittels IBM SPSS Statistics 28 (Fa. IBM Corp., Armonk, NY, USA), MATLAB 9.13 (Fa. The MathWorks Inc., Natick, MA, USA) und SigmaPlot 14 (Fa. Systat Software Inc., San José, CA, USA). In der deskriptiven Statistik wurden Mittelwert (M), Standardabweichung (SD) und Median (Md) verwendet, für die Normalverteilungsprüfung der Shapiro-Wilk-Test. Aufgrund überwiegend nichtnormalverteilter Daten wurden für die Interferenzstatistik nonparametrische Verfahren angewandt: Kruskal-Wallis-Test mit paarweisen Vergleichen und Bonferroni-Korrektur, Spearman-Rangkorrelationen. Das Signifikanzniveau wurde auf 5 % gesetzt. Grafische Boxplots zeigen Median und Perzentile, die Box definiert das 25. und das 75. Perzentil, die Fehlerbalken das 10. bzw. 90. Perzentil; die Ausreißer sind zusammengefasst und zeigen das 5. bzw. 95. Perzentil.

## Ergebnisse

Die Ergebnisse werden nachfolgend für beide ELFRA-Versionen getrennt und jeweils für die Bezugsmaße LA und HA dargestellt. Mediane und Quartile wurden in Boxplots grafisch dargestellt. Es erfolgte zudem ein Vergleich der Mittelwerte mit jenen der Normierungsstichproben entsprechend der dort vorgenommen Gruppierung nach Risikogruppe (erzielte Werte unterhalb des kritischen Werts) und der unauffälligen Kontrollgruppe [13]. Anschließend folgen die Korrelationsanalysen.

### Leistungen im ELFRA-1

Bei LA 12 (M = 15,3; SD = 2,3) lagen 63 % der Teilstichprobe (*n*_*LA12*_ = 99) in der Skala Sprachproduktion (SP) unterhalb des kritischen Wertes von 7 (Md = 5; M = 6,8; *SD* = 6,4), im Vergleich liegt der Mittelwert somit nahe dem der Risikogruppe (M = 6,40). In der Skala Sprachverständnis (SV) lagen 50 % unterhalb des kritischen Werts 17 (Md = 17; M = 32,9; SD = 37,2), die Leistung liegt zwischen der Risikogruppe (M = 24,6) und der Kontrollgruppe (M = 37,2). In der Skala Gesten (G) lagen 31 % unterhalb des kritischen Werts 11 (Md = 15; M = 14,3; SD = 6,7), dies entspricht der Leistung der Kontrollgruppe (M = 14,9). Der kritische Wert 7 wurde in der Skala Feinmotorik (F) von 25 % unterschritten (Md = 10; M = 9,2; SD = 2,9), der Mittelwert liegt deskriptiv über dem der Kontrollgruppe (M = 8,5).

Bei HA 12 (M = 12,9; SD = 1,7) zeigte die Teilstichprobe (*n*_*HA12*_ = 233) deskriptiv deutlich bessere Leistungen. In der Skala SP (Md = 28; M = 41,5; SD = 36,9) unterschritten 12 % und in der Skala SV (Md = 82; M = 85,5; SD = 48,1) 8 % die kritischen Werte. In den beiden Skalen G (Md = 24; M = 23,4; SD = 5,5) und F (Md = 13; M = 12,0; SD = 2,2) lagen alle (100 %) weit oberhalb der kritischen Werte. Alle Mittelwerte der Teilstichprobe lagen deskriptiv weit über denen der Kontrollgruppen (SP M = 11,3; SV M = 37,2; G M = 14,9; F M = 8,5).

**Abb. 1:**
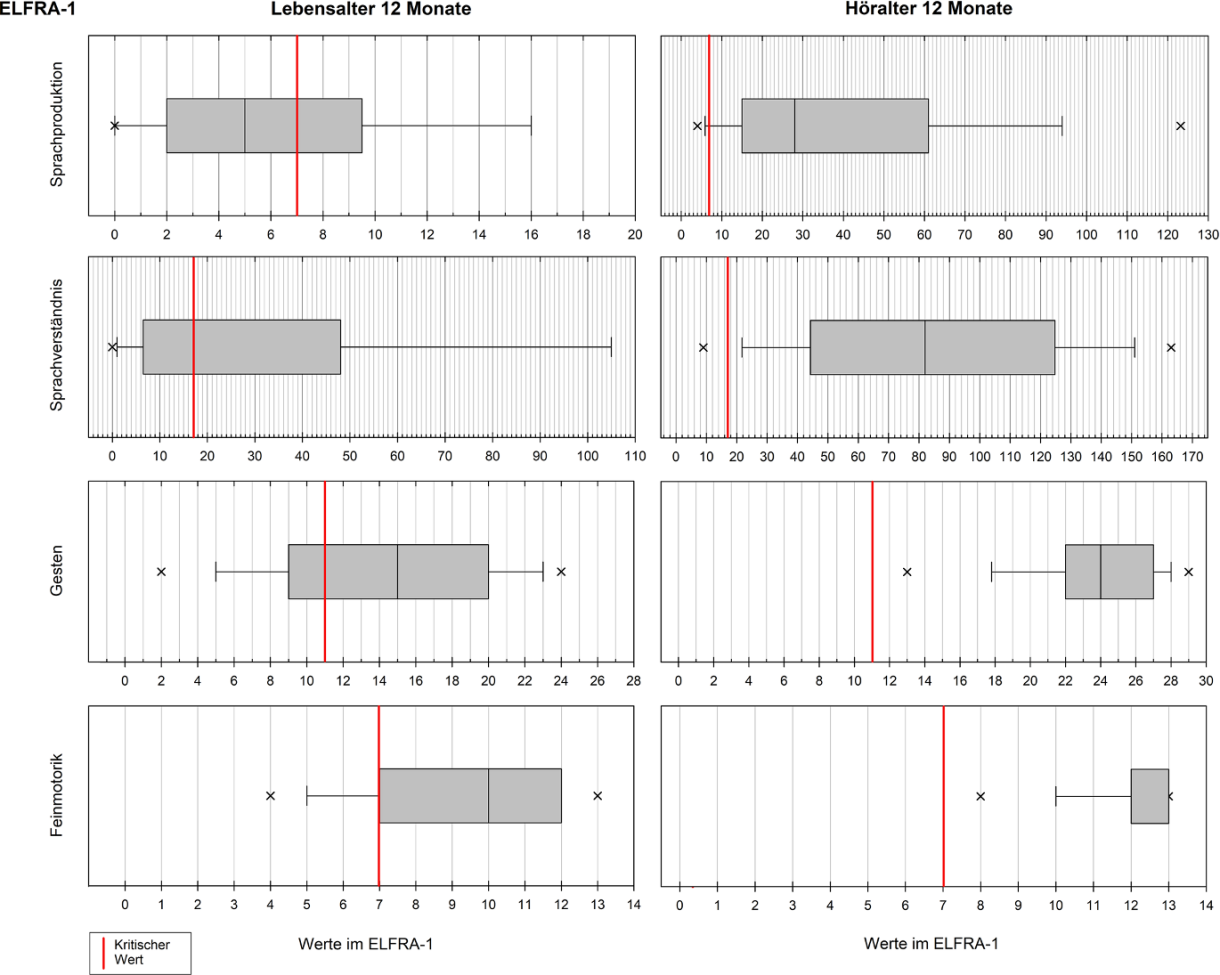
Ergebnisse der Teilstichproben im Elternfragebogen zur Früherkennung von Risikokindern (ELFRA-1) getrennt für Lebensalter 12 Monate (*n*_*LA12*_ = 99) und Höralter 12 Monate (*n*_*HA12*_ = 233), jeweils differenziert für die 4 Skalen mit Angabe der definierten kritischen Werte. *Höhere Werte bedeuten *bessere Leistungen

Die Werte und Verteilungen sind in Abb. 1 dargestellt, getrennt für LA und HA jeweils für die Skalen des ELFRA‑1. Während die Leistungen nach LA in allen 4 Skalen des ELFRA‑1 ähnlich breit innerhalb der Wertebereiche streuten, zeigte sich für das HA eine breite Streuung für die Skalen SP und SV und homogenere Leistungen in den Skalen G und F.

Im Kruskal-Wallis-Test ergaben sich für das LA in den Skalen SP (*p* = 0,295), G (*p* = 0,246) und F (*p* = 0,146) keine signifikanten Unterschiede zwischen Kindern mit monolingual deutschem, bi- oder multilingualem und bimodalem (deutsche Lautsprache und Gebärdensprache) Spracherwerb. Nur in der Skala SV zeigten sich signifikant geringere Leistungen in der bi-/multilingualen Gruppe (*p* < 0,021). Für das HA zeigte die bi-/multilinguale Gruppe signifikant geringere Leistungen gegenüber den beiden anderen Gruppen sowohl in der Skala SP (*p* = 0,005 und *p* = 0,001) als auch in der Skala SV (*p* = 0,001 und *p* = 0,049). Die paarweisen Vergleiche ergaben unabhängig von LA oder HA und unabhängig von den Skalen keine signifikanten Unterschiede zwischen den monolingualen und bimodalen Gruppen.

### Leistungen im ELFRA-2

Bei LA 24 (M = 24,7; SD = 0,9) ergab sich für die Teilstichprobe (*n*_*LA24*_ = 72) in der Skala Produktiver Wortschatz (PW) ein Anteil von 57 % (Md = 40, M = 53,4; SD = 47,3), der den kritischen Wert 50 unterschritt. Im Mittel lag die Leistung näher an jener der Risikogruppe (M = 29,2) als an der Kontrollgruppe (M = 150,3). In der Skala Syntax (Syn) unterschritten 64 % den kritischen Wert 7 (Md = 2,5; M = 6,5; SD = 8,4), in der Skala Morphologie (Mor) 69 % den kritischen Wert 2 (Md = 0; M = 1,6; SD = 2,7). Dies entspricht in beiden Skalen der Leistung der Risikogruppen (Syn M = 6,9; Mor M = 1,4).

Analog zu den Ergebnissen des ELFRA‑1 ergaben sich in allen 3 Skalen für die Teilstichprobe (*n*_*HA24*_ = 161) HA 24 (M = 24,3; SD = 1,2) deskriptiv bessere Leistungen als für LA 24. Unterhalb der kritischen Werte lagen in der Skala PW (Md = 145; M = 136,2; SD = 68,6) 16 %, in der Skala Syn (Md = 23,5; M = 22,2; SD = 12,7) 14 % und in der Skala Mor (Md = 7; M = 7,0; SD = 5,9) 21 %. Tendenziell waren die Leistungsbereiche vergleichbar mit den Mittelwerten der Kontrollgruppen (PW M = 150,3; Syn M = 25,9; Mor M = 9,5).

**Abb. 2:**
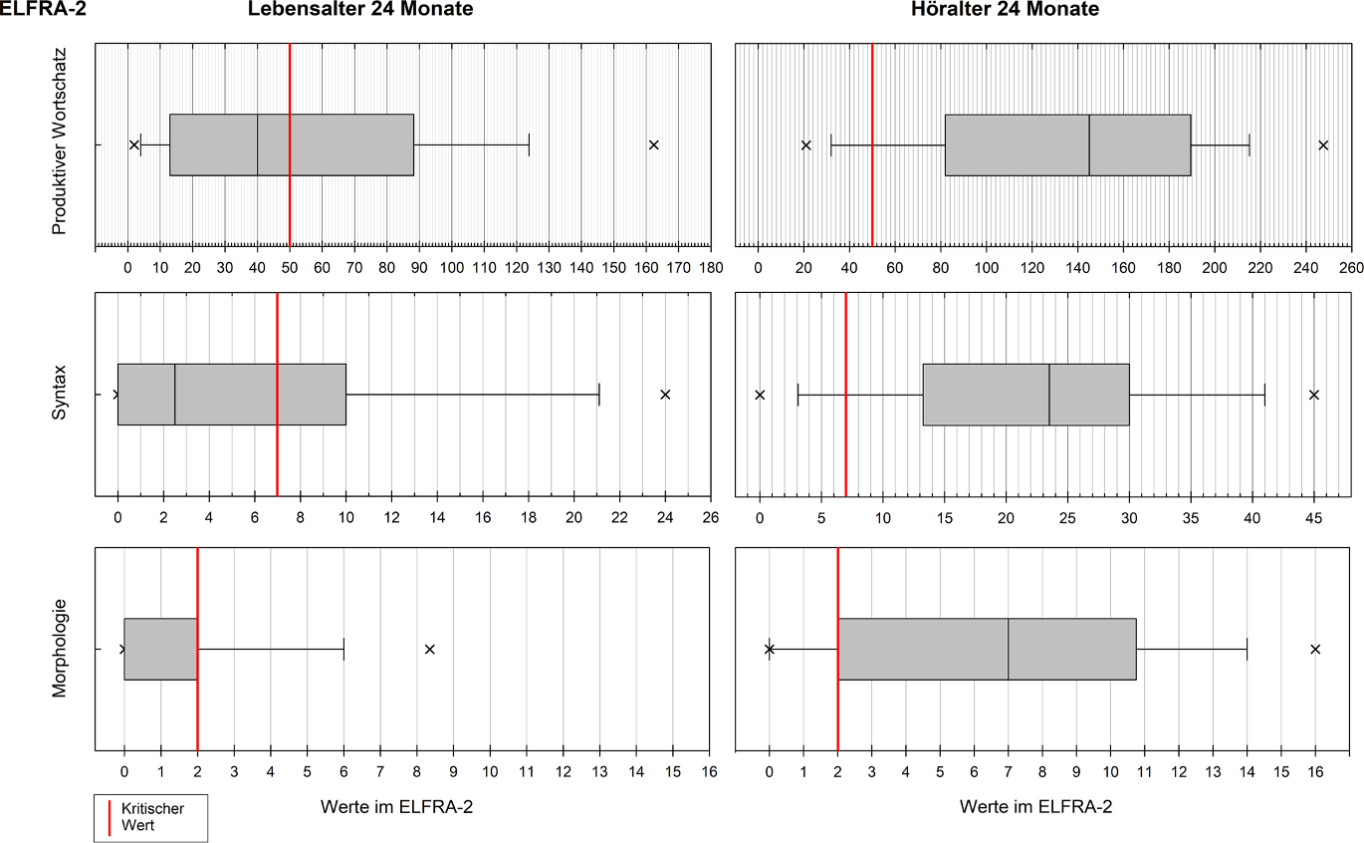
Ergebnisse der Teilstichproben im Elternfragebogen zur Früherkennung von Risikokindern (ELFRA-2) getrennt für Lebensalter 24 Monate (*n*_*LA24*_ = 72) und Höralter 24 Monate (*n*_*HA24*_ = 161), jeweils differenziert für die 3 Skalen mit Angabe der definierten kritischen Werte. *Höhere Werte bedeuten* bessere Leistungen

Die Streuung der Leistungen nach HA erstreckte sich in allen Skalen breit über die jeweiligen Wertebereiche; nach LA liegen die Perzentile 25 bis 75 maximal im ersten Drittel des Wertebereichs. Die Abb. 2 zeigt die Verteilungen und Werte getrennt für LA und HA jeweils für die Skalen im ELFRA‑2.

Keine signifikanten Unterschiede ergaben sich im Kruskal-Wallis-Tests nach LA zwischen den Gruppen monolingual, bi-/multilingual und bimodal (*p* > 0,090). Für das HA zeigten sich dagegen in den paarweisen Vergleichen signifikant geringere Leistungen in der bi-/multilingualen Gruppe; in der Skala PW im Vergleich zu monolingualer (*p* = 0,014) und bimodaler Gruppe (*p* < 0,0001), in den Skalen Syn (*p* *=* 0,004) und Mor (*p* = 0,034) jeweils nur im Vergleich mit der monolingualen Gruppe. Die paarweisen Vergleiche ergaben analog zum ELFRA‑1 unabhängig von LA oder HA und unabhängig von den Skalen keine signifikanten Unterschiede zwischen den monolingualen und bimodalen Gruppen.

### Korrelationen mit dem CI-Versorgungsalter

Mittels Korrelationsanalysen nach Spearman wurden die Leistungen im ELFRA‑1 und ELFRA‑2 jeweils separat für LA und HA auf Zusammenhänge mit dem CI-Versorgungsalter untersucht. Für LA 12 zeigten sich signifikante positive Korrelationen im ELFRA‑1 nur für die Skalen G (rho = 0,38) und F (rho = 0,28), während für LA 24 im ELFRA‑2 alle Skalen (PW rho = −0,38; Syn rho = −0,29; Mor rho = −0,34) signifikant negativ mit dem CI-Versorgungsalter korrelierten. Für HA 24 fanden sich für den ELFRA‑2 dagegen keine Zusammenhänge. Für HA 12 ergaben sich im ELFRA‑1 für die Skalen G (rho = 0,27) und F (rho = 0,39) signifikante positive Korrelationen. Die genauen Koeffizienten für alle analysierten Daten sind in Tab. 3 dargestellt.

**Tab. 3:** Spearman-Rho-Korrelationen zur Ermittlung von Zusammenhängen zwischen dem CI-Versorgungsalter und den Leistungen in den Elternfragebögen zur Früherkennung von Risikokindern (ELFRA) in Abhängigkeit vom Testzeitpunkt (p < 0,05: *; p < 0,01:**, p < 0,001:***).

	Entwicklungsskala	Testzeitpunkt Lebensalter mit CI-Versorgungsalter		Testzeitpunkt Höralter mit CI-Versorgungsalter	
		rho	*p*	rho	*p*
ELFRA‑1	Sprachproduktion	0,20	0,059	0,08	0,277
	Sprachverständnis	−0,03	0,819	−0,02	0,820
	Gesten	0,38***	< 0,001	0,27***	< 0,001
	Feinmotorik	0,28**	0,005	0,39***	< 0,001
ELFRA‑2	Produktiver Wortschatz	−0,38***	< 0,001	0,03	0,710
	Syntax	−0,29*	0,014	−0,04	0,601
	Morphologie	−0,34**	0,004	0,03	0,754

## Diskussion

### Verbale und präverbale Kompetenzen im ELFRA-1

Der ELFRA‑1 erfasst verbale und präverbale Kompetenzbereiche sehr junger Kinder. Die Analyse ergab für die verbalen rezeptiven und produktiven Kompetenzen geringere LA-bezogene Leistungen in der untersuchten Kohorte im Vergleich zur Kontrollgruppe der Normierungsstichprobe. Hinsichtlich der LA-bezogenen gestischen und feinmotorischen Fertigkeiten schlossen dagegen mehr als zwei Drittel zu ihrer normalhörenden Alterskohorte auf. Diese präverbalen Kompetenzen scheinen sich demnach weitestgehend normal zu entwickeln, Befunde von Tait et al. [26] zur präverbalen Kommunikationsentwicklung sehr früh CI-versorgter Kinder stehen damit in Einklang. Bereits der präoperative Entwicklungsstatus von Gestengebrauch und Feinmotorik wurde von Bavin et al. [1] als Prädiktor für die frühe Wortschatzentwicklung CI-versorgter Kinder identifiziert. Inwiefern der ELFRA‑1 hierfür jedoch generell ein geeignetes Erhebungsinstrument darstellt, ist kritisch zu beleuchten: In einer Arbeit von Sachse et al. [23] erwies sich zum einen die prognostische Validität des ELFRA‑1 als unzureichend. Zum zweiten soll laut Fragebogenmanual die Skala Feinmotorik vorrangig der Erfassung des entwicklungsneurologischen Status dienen [13]. Drittens deckt der ELFRA‑1 nicht alle relevanten präverbalen Kompetenzbereiche ab. Unter dem Aspekt der Leistbarkeit von Status- und Verlaufsdiagnostik im Rahmen der CI-Rehabilitation hat sich der ELFRA‑1 jedoch als praktikabel erwiesen und ist seit Jahren zentrumsübergreifend etabliert. Auch die Befunde der vorliegenden Studie unterstützen das Vorgehen, Gestik und Feinmotorik in den frühen prä- und postoperativen Phasen mit dem ELFRA‑1 zu erheben, um im Mindesten einen Eindruck über den Entwicklungsstand präverbaler Teilbereiche zu gewinnen. Um umfassendere Beurteilungen vornehmen zu können, sollten zukünftige Analysen die Ergebnisse weiterer Diagnostikinstrumente einbinden. Für den präverbalen Teilbereich des stimmlich-expressiven Verhaltens im Sinne prosodischer Elemente frühkindlicher Vokalisationen [29] eignet sich der LittlEARS®-Sprachproduktionsfragebogen [28], der LittlEARS®-Hörfragebogen [18] eignet sich in besonderem Maße für den Teilbereich des auditiv-rezeptiven Verhaltens.

Eben dieses auditiv-rezeptive Verhalten entwickelt sich erst durch den auditiven Input über das CI. Dessen Bedeutung wird deutlich bei Betrachtung der Ergebnisse zu den LA-bezogenen verbalen Kompetenzen: In der betreffenden Kohorte war das mittlere CI-Versorgungsalter 10,6 Monate und somit der mittlere zeitliche Abstand zum Testzeitpunkt mit 4,7 Monaten gering. LA-gemäße verbale Leistungen sind demnach an dieser Stelle eher nicht zu erwarten, und es überrascht nicht, dass sich zu diesem frühen Testzeitpunkt auch keine Unterschiede hinsichtlich der Sprachproduktion zwischen Kindern mit mono-, bi-/multilingualem und bimodalem Spracherwerb fanden. Im rezeptiven Bereich zeigte sich dagegen ein Rückstand der bi-/multilingualen Gruppe. Dies könnte sich als früher Hinweis auf spätere sprachbereichsübergreifende Leistungsdefizite interpretieren lassen, eindeutige Aussagen sind an dieser Stelle jedoch nicht zulässig.

Im Vergleich zum LA fallen für das HA die insgesamt weit besseren Ergebnisse auf. Im Mittel erfolgte die Testung 12,9 Monate nach der CI-Versorgung, sodass mit dieser Dauer an Hörerfahrung sowie höherem Lebensalter weiter entwickelte Fähigkeiten zu erwarten sind. Nur noch etwa ein Zehntel erreichte nicht die kritischen Werte in den verbalen Skalen, und deren Mittelwerte sowie die der präverbalen Skalen lagen weit über den Mittelwerten der altersgemäß entwickelten Kontrollgruppe. Auch Mikolajczak et al. [20] untersuchten früh CI-versorgte Kinder und deren Ergebnisse im ELFRA‑1 und fanden ebenfalls unauffällige Werte bezogen auf das HA in allen Skalen des ELFRA‑1 und zudem bessere Leistungen von monolingual gegenüber bilingual aufwachsenden Kindern. Die Ergebnisse der vorliegenden Studie können dies – zumindest für die verbalen Fertigkeiten – bestätigen.

### Verbale Kompetenzen im ELFRA-2

Im ELFRA‑2 werden ausschließlich verbale Kompetenzen erfasst und die LA-bezogene Analyse zeigte, dass etwas mehr als die Hälfte der Kinder die kritische Wortschatzmarke nicht erreichte. Während im lexikalischen Bereich der Mittelwert zwar über dem der Risikogruppe, jedoch weit unter dem der Kontrollgruppe lag, entsprachen die Leistungen im morphosyntaktischen Bereich eindeutig denen der Risikogruppe. In der Wortschatzentwicklung scheinen die Kinder demnach schneller an ihre normalhörende Altersgruppe aufzuschließen, was sich mit Befunden anderer Studien deckt [2, 4, 11]. Analog zum ELFRA‑1 fielen die Leistungen nach HA im ELFRA‑2 erwartungsgemäß weitaus besser als nach LA aus, weit mehr als drei Viertel zeigten hier verbale Kompetenzen oberhalb der kritischen Werte.

Ein positiver Effekt von monolingualem gegenüber bi-/multilingualem Spracherwerb lässt sich annehmen, und dieser scheint zunehmend stärker zu werden, je besser die verbalen Kompetenzen ausgebildet sind. Hinweise darauf liefern die Gruppenvergleiche, die zeigten, dass in der HA-bezogenen Analyse signifikante Unterschiede sowohl im lexikalischen als auch im morphosyntaktischen Bereich bestanden. Die LA-bezogene Analyse hingegen ergab keine bedeutsamen Unterschiede. Diese Befunde sind vereinbar mit denen einer Studie mit deutschsprachigen Kindern mit CI oder Hörgerät, in der die Autoren über deutlich geringere Sprachkompetenzen bei bilingualen Kindern berichteten [16]. Des Weiteren weisen die Ergebnisse der Vergleichsanalysen darauf hin, dass ein bimodaler Spracherwerb keinen Einfluss auf die mit den ELFRA erfassten vor- und lautsprachlichen Kompetenzen nimmt.

### Relevanz des CI-Versorgungsalters

Ein Zusammenhang zwischen CI-Versorgungsalter und frühen Sprachleistungen wurde mittels Rangkorrelationsanalysen geprüft. Die Ergebnisse legen nahe, dass sich ein positiver Effekt einer zeitlich sehr frühen CI-Versorgung erst bei einem Lebensalter von etwa 2 Jahren zeigt. Dies gilt zumindest für die verbalen Kompetenzen, während sich für die präverbalen Kompetenzen bereits im ELFRA‑1 bedeutsam bessere LA- und HA-bezogene Leistungen mit jüngerem CI-Versorgungsalter fanden. Der Fortschritt der gestischen und feinmotorischen Entwicklung scheint demnach mit dem CI-Versorgungsalter zusammenzuhängen. Betrachtet man im speziellen die verbalen Kompetenzen im ELFRA‑2, so fällt auf, dass diese lediglich für die LA-bezogenen Leistungen mit dem CI-Versorgungsalter korreliert waren. Dieser Befund zeigt, dass durch eine sehr frühe CI-Versorgung ein Aufschließen zu normalhörenden Kindern im Sinne des Erreichens von LA-adäquaten Sprachleistungen begünstigt wird. Befunde anderer Studien [3, 7] zur Bedeutung einer sehr frühen CI-Versorgung für die Sprachentwicklung werden damit bestätigt.

### Relevanz des Bezugsmaßes

Die separaten Analysen hinsichtlich der Bezugsmaße LA und HA zeigten für beide Versionen ELFRA‑1 und ELFRA‑2 erwartungsgemäß klare Unterschiede auf. Die LA-bezogene Auswertung spiegelt die tatsächlichen Leistungen der untersuchten Kohorten im Vergleich zu den normalhörenden Normierungsstichproben wider; die Kohorten schnitten in diesem Vergleich tendenziell unterdurchschnittlich ab. In den HA-Analysen lagen die Leistungen größtenteils weit oberhalb der Norm. Dies spricht dafür, dass die HA-bezogene Sprachdiagnostik den Entwicklungstand bereits in den sehr frühen Spracherwerbsphasen zu überschätzen bzw. zu verzerren scheint. Unter diesem Aspekt ist zudem bemerkenswert, dass die HA-bezogenen sprachlichen Kompetenzen nicht im Zusammenhang mit dem CI-Versorgungsalter stehen.

Für CI-versorgte Kinder, die den Einschlusskriterien der vorliegenden Studie entsprechen, ergibt sich daraus die eindeutige Empfehlung, das LA als Bezugsmaß in der frühen Sprachdiagnostik dem HA vorzuziehen. Eine LA-bezogene Diagnostik ermöglicht die quantitative und qualitative Feststellung von Ressourcen und Entwicklungsrückständen und erlaubt somit eine eindeutige Beurteilung des reellen Entwicklungsstands über alle präverbalen und verbalen Bereiche hinweg. Insbesondere während der postoperativen Rehabilitation ist dies wichtig für die Festlegung von Therapiezielen und -inhalten.

Eine frühere Studie [10], die sich mit der Aussagekraft von Bezugsmaßen in der Sprachdiagnostik bei CI-versorgten Kindern befasste, kam zu dem Schluss, dass das CI-Versorgungsalter ein wichtiges Kriterium bei der Auswahl des Bezugsmaßes sein sollte: Da sich mit früherem CI-Versorgungsalter das HA dem LA annähert bzw. sich die auditive Deprivationsphase zeitlich verringert, ist eine geringere Entwicklungslücke nach der CI-Versorgung zu schließen. Die Wahrscheinlichkeit, dass der Spracherwerb – auch bereits in frühen Phasen – altersgemäß verläuft, ist dadurch höher, sofern keine zusätzlichen Risiken bestehen, die die Entwicklung ungünstig beeinflussen oder hemmen könnten. Als solche Risiken sind zum einen das Vorliegen von Komorbiditäten oder zusätzlicher Entwicklungsbeeinträchtigungen [5, 12] zu nennen, ebenso wie ein bi-/multilingualer Spracherwerb [16, 27]. Letzteres bestätigen auch die Vergleichsanalysen der vorliegenden und der früheren Studie [10]. Besteht aufgrund ungünstiger Spracherwerbsbedingungen ein erheblicher allgemeiner Entwicklungsrückstand, können bereichspezifische Defizite und Ressourcen anhand des LA unter Umständen nicht mehr ausreichend differenziert erfasst und beurteilt werden. Dadurch kann auch die Therapieplanung erschwert sein. In diesen Fällen kann das HA als Bezugsmaß in der Diagnostik geeigneter sein und konkretere Ergebnisse für die individuelle Therapiegestaltung liefern. Empirische Studien hierzu stehen jedoch noch aus.

### Referenzwerte für CI-versorgte Kinder

Im deutschsprachigen Raum existieren für CI-versorgte Kinder keine explizit normierten sprachspezifischen Erhebungsverfahren, sodass in der Diagnostik Tests und Fragebögen verwendet werden müssen, die für normalhörende Kinder konstruiert wurden. Ein Anliegen der Multizenterstudie war es deshalb, anhand größerer Kohorten CI-versorgter Kinder Referenzwerte für einzelne Erhebungsverfahren bereitstellen zu können. Die Daten der vorliegenden Analyse können in diesem Sinne eingeschränkt verwendet werden: Die Daten sind spezifisch repräsentativ für Kinder, die bis zu einem maximalen Lebensalter von 48 Monaten bilateral CI-versorgt wurden und keine Zusatzbeeinträchtigungen aufwiesen. Erfüllt ein Kind diese Kriterien, können die ermittelten und anhand von Boxplots dargestellten Quartile und Perzentile als Vergleichswerte für individuell erreichte Werte herangezogen werden.

Für Kinder mit Zusatzbeeinträchtigung gilt dies jedoch nicht. Wenn man bedenkt, dass bei 20–40 % neben der Hörminderung Zusatzbeeinträchtigungen vorliegen [24], wird deutlich, welch großer Anteil an Kindern unberücksichtigt bleibt. Zukünftige Arbeiten sollten daher gezielt diese Gruppe in den Fokus nehmen.

Auch die Anwendung als Referenzwerte für Kinder mit bi-/multilingualem Spracherwerb ist stark limitiert: Nach aktuellem Forschungsstand gilt für diese Gruppe grundsätzlich der Einsatz von monolingual deutschen Sprachdiagnostikerfahren als ungeeignet [25]. Darüber hinaus wären weitere sozioökonomische Variablen mit zu erheben und in Analysen zu integrieren, welche auch im Kontext bilingualer bzw. bimodaler Spracherwerbsbedingungen relevant sind. Hier bedarf es spezifischer Forschung, um Einflussfaktoren eindeutig identifizieren zu können; die Ergebnisse der vorliegenden Arbeit können daher lediglich als Trends betrachtet werden.

Eine weitere Limitation ist der geringe Umfang der jeweiligen Teilstichproben je nach Version des ELFRA sowie separiert für LA und HA. Auch wenn die Testpopulation merkmalsbezogen relativ homogen ist, so wären für eine valide Normierung größere Stichprobenumfänge und Prüfungen der Verteilungseigenschaften der Stichprobenmerkmale nötig. Positiv – im Sinne der Testtheorie – zu werten ist die Repräsentativität der Stichprobe an inkludierten Kindern hinsichtlich der bundesweit verteilten Regionen durch die Verortung der beteiligten CI-Centren.

## Ausblick

Aufgrund der heute etablierten CI-Versorgung möglichst innerhalb des ersten Lebensjahres ist die Erfassung von Kompetenzen früher Phasen der Sprachentwicklung unter Einbezug präverbaler Fertigkeiten von hoher Bedeutung. In der Diagnostik und Leistungsbeurteilung sollte bevorzugt das Lebensalter des CI-versorgten Kindes als Bezugsmaß eingesetzt werden. Die Verwendung des Höralters sollte sich auf Kinder mit ungünstigeren Spracherwerbsbedingungen (z. B. bi-/multilingualem Spracherwerb, Zusatzbeeinträchtigung und späte CI-Versorgung nach dem zweiten Lebensjahr) begrenzen.

Die mittels der Kohortenanalysen ermittelten Perzentile können eingeschränkt als Referenzwerte dienen, um den individuellen Leistungsstand im Vergleich zu anderen CI-versorgten Kindern besser einschätzen zu können.

Weitere multizentrische Studien sind anzustreben, um den Datenpool zu vergrößern und darüber hinaus normierbare Referenzwerte für spezifische Versorgungsgruppen und Alterskohorten zu generieren.

## Fazit für die Praxis

- In der Sprachdiagnostik bei sehr früh mit einem Cochleaimplantat (CI-)versorgten Kindern ohne Zusatzbeeinträchtigung sollten neben den verbalen auch präverbale Kompetenzen ermittelt werden, hierfür eignet sich für Teilbereiche das Verfahren Elternfragebögen zur Früherkennung von Risikokindern (ELFRA).
- Für diese Zielgruppe ist das Lebensalter als Bezugs- und Beurteilungsmaß dem Höralter vorzuziehen.
- Bei diesen Patienten können die dargestellten Perzentile für den ELFRA‑1 und ELFRA‑2 und unterteilt nach Lebensalter und Höralter als Referenzdaten zur unterstützenden Einschätzung individueller Leistungen herangezogen werden.
